# Designation of optimal reference strains representing the infant gut bifidobacterial species through a comprehensive multi‐omics approach

**DOI:** 10.1111/1462-2920.16205

**Published:** 2022-10-07

**Authors:** Federico Fontana, Giulia Alessandri, Chiara Tarracchini, Massimiliano Giovanni Bianchi, Sonia Mirjam Rizzo, Leonardo Mancabelli, Gabriele Andrea Lugli, Chiara Argentini, Laura Maria Vergna, Rosaria Anzalone, Giulia Longhi, Alice Viappiani, Giuseppe Taurino, Martina Chiu, Francesca Turroni, Ovidio Bussolati, Douwe van Sinderen, Christian Milani, Marco Ventura

**Affiliations:** ^1^ Laboratory of Probiogenomics, Department of Chemistry, Life Sciences, and Environmental Sustainability University of Parma Parma Italy; ^2^ GenProbio srl Parma Italy; ^3^ Laboratory of General Pathology, Department of Medicine and Surgery University of Parma Parma Italy; ^4^ Microbiome Research Hub University of Parma Parma Italy; ^5^ APC Microbiome Institute and School of Microbiology, Bioscience Institute National University of Ireland Cork Ireland

## Abstract

The genomic era has resulted in the generation of a massive amount of genetic data concerning the genomic diversity of bacterial taxa. As a result, the microbiological community is increasingly looking for ways to define reference bacterial strains to perform experiments that are representative of the entire bacterial species. Despite this, there is currently no established approach allowing a reliable identification of reference strains based on a comprehensive genomic, ecological, and functional context. In the current study, we developed a comprehensive multi‐omics approach that will allow the identification of the optimal reference strains using the *Bifidobacterium* genus as test case. Strain tracking analysis based on 1664 shotgun metagenomics datasets of healthy infant faecal samples were employed to identify bifidobacterial strains suitable for in silico and in vitro analyses. Subsequently, an ad hoc bioinformatic tool was developed to screen local strain collections for the most suitable species‐representative strain alternative. The here presented approach was validated using in vitro trials followed by metagenomics and metatranscriptomics analyses. Altogether, these results demonstrated the validity of the proposed model for reference strain selection, thus allowing improved in silico and in vitro investigations both in terms of cross‐laboratory reproducibility and relevance of research findings.

## INTRODUCTION

Historically, the term ‘type strain’ is employed to denote bacterial clonal descendants of the first isolated member of a novel species, also indicated as ‘nomenclatural type’ (Parker et al., 2019). Consequently, type strains were commonly used to perform pairwise comparisons for the assignment of bacterial names, acting as a milestone for taxonomical definition of a microbial taxon (Lapage et al., 1992).

Nevertheless, type strains of most known microbial species may not represent the most suitable candidates to represent their species from an ecological, genomic and functional viewpoint (Kyrpides et al., 2014). For these reasons, the rapid progress in investigating the microbial composition of the human gut microbiota has prompted the need to identify a model strain to be used for experiments to generate results that are representative of the entire bacterial species, thus defining the concept of reference strain (Parker et al., 2019). However, the reference strain definition is somewhat general, indicated as a strain used in comparative studies or any strain derived from a recognized culture collection (Kyrpides et al., 2014; Parker et al., 2019). It is therefore important to identify suitable and relevant microbial reference strains to represent key bacterial species of the human gut microbiota by employing a comprehensive multi‐omics approach.

Among the hundreds of different bacterial species inhabiting the human intestine, members of the genus *Bifidobacterium* have received substantial research consideration in recent decades. Bifidobacteria are among the first colonizers of the infant gut, persisting at high abundance at least until the weaning phase, while certain species/strains of this genus are claimed to exert multiple beneficial effects on host health (Alessandri et al., 2019, 2021; Bottacini et al., 2017; Hidalgo‐Cantabrana et al., 2017; Milani et al., 2016; Rivière et al., 2016; Turroni, Milani, Duranti, Mahony, et al., 2018). In this context, various studies have investigated the bifidobacterial community present in the infant intestine through metagenomic analyses of faecal samples, revealing that *Bifidobacterium bifidum*, *Bifidobacterium breve*, *Bifidobacterium longum* subsp. *infantis* and *B. longum* subsp. *longum* represent the most prevalent and abundant bifidobacterial species of the ‘infant‐like’ gut microbiota (Arboleya et al., 2016; Duranti et al., 2017; Milani et al., 2015; Tarracchini et al., 2022; Turroni et al., 2021; Turroni, Milani, Duranti, Ferrario, et al., 2018). Furthermore, scientific interest in bifidobacteria has led to extensive isolation efforts that now allow access to a large number of strains and their corresponding genomic sequences (Saturio et al., 2021; Turroni et al., 2022), rendering this genus a suitable example to perform phylogenomic studies.

There is currently no established approach to appropriately assign reference strains on the basis of genetic/genomic evidence, which should more accurately represent the typical biological features that characterize each bifidobacterial species. Notably, in most cases, selection of bifidobacterial strains for in vitro and in vivo studies is not based on current physiological, ecological and genetic knowledge, possibly causing inaccurate or confusing insights (Arboleya et al., 2016; Duranti et al., 2017; Milani et al., 2015; Turroni, Milani, Duranti, Ferrario, et al., 2018). In fact, several bifidobacterial comparative genomic analyses have reported remarkable intra‐species genetic variability, principally due to the presence of a high average number of truly unique genes, that is, genetic sequences encoded by a single genome, within a single bifidobacterial species. In addition, these highly variable genetic traits are responsible for metabolic and physiological differences among strains of a given species, which may complicate experimental consistency of traits that could be applied to the designation of valuable reference strains for each bifidobacterial specie (Bottacini et al., 2018; Lugli et al., 2020; Lugli, Duranti, Albert, et al., 2019; Lugli, Mancino, Milani, et al., 2019; Tarracchini et al., 2021).

To address this issue, we assessed the abundance of publicly available strains belonging to bifidobacterial species harboured by the infant gut through a very detailed strain‐genomic reconstruction of the infant gut microbiomes. Strain profiling data were then coupled with genomic and comparative genome analyses to define the most representative strains of each bifidobacterial species in the collected infant gut microbiomes. We then developed a bioinformatic tool, here referred to as RefBifSelector, which performs a phylogenetically based rapid screening of the local bifidobacterial strain collections, allowing the identification of the ‘most suitable and locally available bifidobacterial strain(s)’ based on the closest genomic relative to the newly assigned reference bifidobacterial strains. The validity of the identified novel representative bifidobacterial model strains in terms of microbe–microbe and microbe–host interactions was investigated by means of metagenomic and metatranscriptomic methods. The latter approaches were applied using in vitro models simulating real‐life settings involving (i) co‐occurrence with complex bacterial communities encompassing other members of the infant gut microbiota and (ii) molecular interactions with human intestinal cells.

## EXPERIMENTAL PROCEDURES

### Dataset selection

All publicly available datasets corresponding to infant faecal samples sequenced through a shotgun metagenomics approach were selected and downloaded from the NCBI SRA repository using NCBI SRA Toolkit 2.11.0 faster‐dump (Leinonen et al., 2011). Specifically, only datasets processed through Illumina sequencing technology were retained to achieve high quality and coverage data. Additionally, only shotgun metagenomic datasets belonging to healthy infants and full‐term infants with age ranging from a few days to 3 years of life and not having undergone drug, probiotic and/or prebiotic treatments were included in this study. Conversely, no exclusion criteria based on diet, mode of delivery or geographical origin were applied for the selection of the final infant cohort.

### Shotgun metagenomics dataset analysis

To analyse only high‐quality SRA samples, each SRA was subjected to a filtering step to remove low‐quality reads (minimum mean quality score 20, window size 5, quality threshold 25 and minimum length 100) using the fastq‐mcf script (https://github.com/ExpressionAnalysis/ea‐utils/blob/wiki/FastqMcf.md). Collected filtered reads were then taxonomically classified through the METAnnotatorX2 pipeline (Milani et al., 2021), using the up‐to‐date RefSeq (genome) database retrieved from the NCBI. Metagenome‐assembled genomes (MAGs) were reconstructed through the METAnnotatorX2 pipeline (Milani et al., 2021) and (meta)SPAdes software (Bankevich et al., 2012). Reconstructed contig >5 kbp were retained and taxonomically classified in the same manner as for filtered reads.

### Bifidobacterial strain selection

To create species‐specific databases comprising bifidobacterial species typical of the infant gut microbiota, filtered reads from online shotgun metagenomics datasets were subjected to whole metagenome assembly using SPAdes v3.14 (Bankevich et al., 2012) with default parameters and the metagenomic flag option (‐meta) together with minimum k‐mer sizes of 21, 33, 55, and 77, as previously described (Lugli et al., 2021; Lugli, Duranti, Milani, et al., 2019; Lugli, Milani, Duranti, et al., 2019). For short reads, reconstructed contig sequences were taxonomically classified based on their sequence identity using megablast against the same RefSeq database (Agarwala et al., 2018). Furthermore, in addition to bifidobacterial genomes reconstructed from shotgun metagenomic datasets, publicly available genomes belonging to *Bifidobacterium* species typical of the infant gut microbiota were selected from the NCBI genome list. In detail, only genome sequences with a genome coverage higher than 30‐fold and containing less than 100 contigs were considered.

### StrainGST‐based creation of bifidobacterial species‐specific databases

The genomes of the selected bifidobacterial strains, both those reconstructed from online datasets and those publicly available and new isolates, were used as input in the StrainGE employing StrainGST analysis (van Dijk et al., 2021) with standard parameters to identify possible redundant genomes among those selected for database creation. In this context, to remove genomes with sequence identity >99% from final databases, straingGST function ‘straingst kmersim’ first calculated Jaccard similarity between k‐mer profiles of each genome pair. K‐mer profiles evaluate the distribution and prevalence of short sequences, that is, k‐mers, across the genomic sequences. This heuristic approach, extensively used in modern genomic and metagenomic analyses, provides a marked reduction in computing time and computational resources needed (Bernard et al., 2018). Subsequently, the function ‘straingst cluster’ was used to remove all genomes which shared 99% of the k‐mers with another genome already included in the database. Additionally, straingGST was exploited to generate clusters of similar genomes with Jaccard similarity between two k‐mer sets higher than 0.90, selecting only the optimal one inside each cluster. Hierarchical clustering (HCL) analysis was performed through further neighbourhood, Pearson correlation and maximum distance. RefBif‐*IS* was then selected using a specific index, called AxP, defined as [the average ANI value of genomes constituting the same HCL] * [prevalence score of the strain in the IGMC] * [100].

### Ethical statement

The study protocol was approved by the Ethical Committee of the ‘Azienda Unità Sanitaria Locale di Reggio Emilia – IRCCS’ in Reggio Emilia, Italy. A signed informed consent was obtained from the legally authorized representative of the infant enrolled in this study.

### Experimental set up for infant gut microbiota stabilization

A faecal sample from a 3‐year‐old healthy infant who had not been treated with antibiotics, prebiotics, or probiotics in the previous 3 months was collected immediately after defecation and transported under anaerobic conditions to the laboratory where it was immediately processed. Specifically, upon receipt, the faecal sample was transferred into an anaerobic chamber and immobilized in 1–2‐mm‐diameter gel beads composed of 2.5% (w/v) gellan gum, 0.25% (w/v) xanthan gum, and 0.2% (w/v) sodium citrate, as previously described (Cinquin et al., 2004; Le Blay et al., 2010; Pham et al., 2019).

The fermentation medium was based on the composition designed to mimic the infant gut environment as previously described (Zihler Berner et al., 2013). Specifically, the medium contained the following components: 5 g L^−1^ starch, 2 g L^−1^ pectin, 1 g L^−1^ guar gum, 4 g L^−1^ mucin from porcine stomach, 2 g L^−1^ xylan, 2 g L^−1^ arabinogalactan, 1 g L^−1^ inulin, 3 g L^−1^ casein, 5 g L^−1^ peptone water, 5 g L^−1^ tryptone, 0.4 g L^−1^ bile salts, 0.005 g L^−1^ FeSO_4_, 4.5 g L^−1^ NaCl, 0.5 g L^−1^ KH_2_PO_4_, 0.61 g L^−1^ MgSO_4_, 0.1 g L^−1^ CaCl_2_, 1.5 g L^−1^ NaHCO_3_, 0.8 g L^−1^ cysteine, 0.2 g L^−1^ MnCl_2,_ 0.05 g L^−1^ hemin, and 1 ml Tween 80 (Macfarlane et al., 1998; Zihler Berner et al., 2013). Final pH was adjusted to 6.8, while, after autoclaving, a 0.2 μm filter‐sterilized vitamin solution (1 ml L^−1^) was added to the medium (Pham et al., 2019). The nutritive medium was freshly prepared daily, autoclaved, and stored at 4°C under stirring until use.

The fermentation setup consisted of a bioreactor (Solaris Biotech Solutions, Italy) with a working volume of 400 ml inoculated with 40 ml (10% v/v) faecal beads. Fermentation started in a batch mode by aseptically replacing spent medium with fresh medium every 12 h for 3 days, after which the fermentation was switched to a continuous mode by feeding the bioreactor with fresh medium at flow rates of 66 ml h^−1^ to obtain a mean retention time of 6 h, as previously described (Doo et al., 2017). Stabilization of the infant gut microbiota was performed under anaerobic conditions, while temperature was set at 37°C, stirring speed at 180 rpm, and pH was maintained automatically at 6.8 by adding 2.5 M NaOH. The fermentation process was carried out for a total of 10 days.

### Bifidobacterial cultivation in a gut‐simulated environment

To evaluate the in vitro ability of bifidobacterial strains selected through the RefBifSelector tool among our repository to grow in an intestinal environment, *B. adolescentis* 713B, *B. bifidum* PRL2010, *B. breve* 1895B, *B. dentium* 181B, *B. longum* subsp. *longum* 39B and *B. pseudocatenulatum* 1896B, as well as the type strains and the identified sub‐optimal strains for each selected bifidobacterial species were in batch cultivated in a complex medium mimicking the infant intestine (Macfarlane et al., 1998) together with the abovementioned stabilized gut microbial community of a 3‐year‐old infant.

Specifically, bifidobacterial strains were revitalized from glycerol‐based stock in MRS broth medium supplemented with 0.05% (w/v) l‐cysteine hydrochloride at 37°C under anaerobic conditions. Subsequently, bifidobacterial strains together with the 10‐day stabilized infant gut microbial community were singularly inoculated in 45 ml of culture medium mimicking the infant intestinal environment (Macfarlane et al., 1998; Zihler Berner et al., 2013) to obtain a final inoculum of 10^5^ cells ml^−1^ of a specific bifidobacterial strain and 10^7^ cells ml^−1^ of the stabilized infant gut microbial community. For each experiment, an aliquot of culture was collected at three different time points, that is, 6, 12, and 24 h after the inoculum and conserved at −20°C until they were processed for DNA extraction and flow cytometry‐based bacterial total count. Batch cultures were incubated in an anaerobic chamber (2.99% H_2_, 17.01% CO_2_ and 80% N_2_) (Concept Ruskinn) at 37°C.

### DNA extraction and shallow shotgun sequencing

Each aliquot of every single obtained batch fermentation, together with the control sample, that is, the 10‐day stabilized infant gut microbial community were subjected to DNA extraction using the QIAmp DNA stool mini kit following the manufacturer's instructions (Qiagen, Germany). The extracted DNA was prepared using the Illumina Nextera XT DNA Library Preparation Kit and following the Illumina NexteraXT protocol. Specifically, DNA samples were enzymatically fragmented, barcoded, and purified using magnetic beads. Subsequently, samples were quantified using a fluorometric Qubit quantification system (Life Technologies, USA), then loaded on a 2200 Tape Station Instrument (Agilent Technologies, USA) and normalized to 4 nM. Paired‐end sequencing was performed using an Illumina MiSeq sequencer with MiSeq Reagent Kit v3 (Illumina Inc., San Diego, USA). Taxonomic reconstruction of shallow shotgun data was performed as described above for shotgun metagenomic data analysis.

### Evaluation of bacterial cell density by flow cytometry

For total cell counts, each culture replicate was 100,000 times diluted in physiological solution (phosphate‐buffered solution [PBS]). Subsequently, 1 ml of the obtained bacterial cell suspension was stained with 1 μl of SYBR®Green I (ThermoFisher Scientific, USA) (1:100 dilution in dimethylsulfoxide; Sigma, Germany), vortex‐mixed and incubated at 37°C in the dark for at least 15 min before measurement. All enumeration experiments were carried out through an Attune NxT flow cytometry (ThermoFisher Scientific, Waltham, MA, USA) equipped with a blue laser set at 50 mW and tuned at an excitation wavelength of 488 nm. Multiparametric analyses were performed on both scattering signals, that is, forward scatter (FSC) and side scatter (SSC), while SYBR Green I fluorescence was detected on the BL1 530/30 nm optical detector. Cell debris was excluded from acquisition analysis by setting a BL1 threshold. In addition, the gated fluorescence events were evaluated on the forward‐sideways density plot to exclude remaining background events and to obtain an accurate microbial cell count. All data were statistically analysed with the Attune NxT flow cytometry software.

### Human cell line culture

Human colorectal carcinoma‐derived Caco‐2 cells (purchased from ATCC) and HT29‐MTX (kindly provided by Prof. Antonietta Baldi, University of Milan), that is, a human colon carcinoma‐derived, mucin‐secreting goblet cell line, were cultured in Minimum Essential Medium (MEM) and Dulbecco's Modified Eagle's medium (DMEM) with high glucose (4.5 g L^−1^) and 10 mM of sodium pyruvate, respectively, as previously described (Doo et al., 2017). In addition, both media were supplemented with 10% fetal bovine serum (FBS), 2 mM glutamine, 100 g ml^−1^ streptomycin, and 100 U ml^−1^ penicillin. Cultures were maintained at 37°C in a humidified atmosphere of 5% CO_2_ in air in 10‐cm dishes and passaged three times a week. Subsequently, a mixed suspension of Caco‐2 and HT29‐MTX cells (7:3) was seeded in DMEM + FBS at a density of ≈10^5^ cells cm^−2^ into cell culture inserts with membrane filters (pore size 0.4 μm) for Falcon 24‐well‐multitrays (Becton, Dickinson & Company, Franklin Lakes, NJ, USA), and cultured for 21 days with a medium replacement every 3 days until a tight monolayer was formed (TEER > 600 Ω • cm^2^).

### Human cell‐monolayers and bifidobacteria

After 21 days from seeding, the culture medium of the 24‐well plates was replaced with fresh antibiotic‐free DMEM. Subsequently, bifidobacterial cells (final concentration 10^8^ cells ml^−1^) were added to Caco‐2/HT29‐MTX cell monolayers, as previously described (Turroni et al., 2014). The 24‐well plates were then incubated in 5% CO_2_ at 37°C for 4 h. After this period of incubation, bacterial cells were recovered in RNA later and stored at −80°C until processing.

For these experiments, each bifidobacterial strain, that is, *B. bifidum* PRL2010, *B. breve* 1895B, *B. longum* subsp. *longum* 39B, and *B. pseudocatenulatum* 1896B, together with the Type Strains and the sub‐optimal strains of the same bifidobacterial species, were grown in MRS broth in anaerobic chamber at 37°C. Once they reached the exponential phase of growth (0.6 < OD_600nm_ < 0.8), cells were enumerated through Thoma cell counting chamber (Herka), possibly diluted to reach a final concentration of 10^8^ cells ml^−1^, washed in PBS, resuspended in DMEM without antibiotics, and seeded on Caco2/HT29‐MTX cell monolayers. Furthermore, for each bifidobacterial strain, a control sample, that is, the bifidobacterial strain resuspended in DMEM and maintained under the same incubation conditions of the 24‐well plates without any contact with human cell lines, was obtained.

### RNA extraction

Total RNA of each considered condition was isolated using a previously described method (Milani et al., 2020; Turroni et al., 2016). Briefly, bifidobacterial cell pellets were resuspended in 1 ml of QIAzol lysis reagent (Qiagen) in a sterile tube containing glass beads (Merk, Germany). Cells were lysed by alternating 2 min of stirring the mix on a Precellys 24 homogenizer (Bertin instruments, France) with 2 min of static cooling in ice. These steps were repeated for three times. The lysed cells were centrifuged at 12,000 rpm for 15 min, and the upper phase was recovered. The RNA samples were then purified using the RNeasy minikit (Qiagen) following the manufacturer's instruction. RNA concentration and purity were evaluated using a spectrophotometer (Eppendorf, Germany).

### RNA sequencing analysis

For RNA sequencing, total RNA (from 100 ng to 1 μg) was treated to remove rRNA by using the QIAseq FastSelect—5S/16S/23S following the manufacturer's instructions (Qiagen). The yield of rRNA depletion was checked by using a 2200 TapeStation (Agilent Technologies, USA). Then, a whole transcriptome library was constructed using the TruSeq Stranded mRNA Sample preparation kit (Illumina). Samples were loaded into a NextSeq high‐output v2 kit (150 cycles) (Illumina) as indicated by the technical support guide. The obtained reads were filtered to remove low‐quality reads (minimum mean quality 20 and minimum length 150 bp) as well as any remaining ribosomal loci using the METAnnotator X2 pipeline (Milani et al., 2021) Subsequently, the retained reads were aligned to the specific reference genome through Bowtie2 software (Langdon, 2015). Analysis of the RPKM values was performed through Artemis using the formula RPKM (Reads Per Kilobase Million) = numReads/(geneLength/1000 * totalNumReads/1000000) (Mortazavi et al., 2008).

## RESULTS AND DISCUSSION

### Ecological and phylogenomic‐driven identification of optimal reference model strains

**FIGURE 1:**
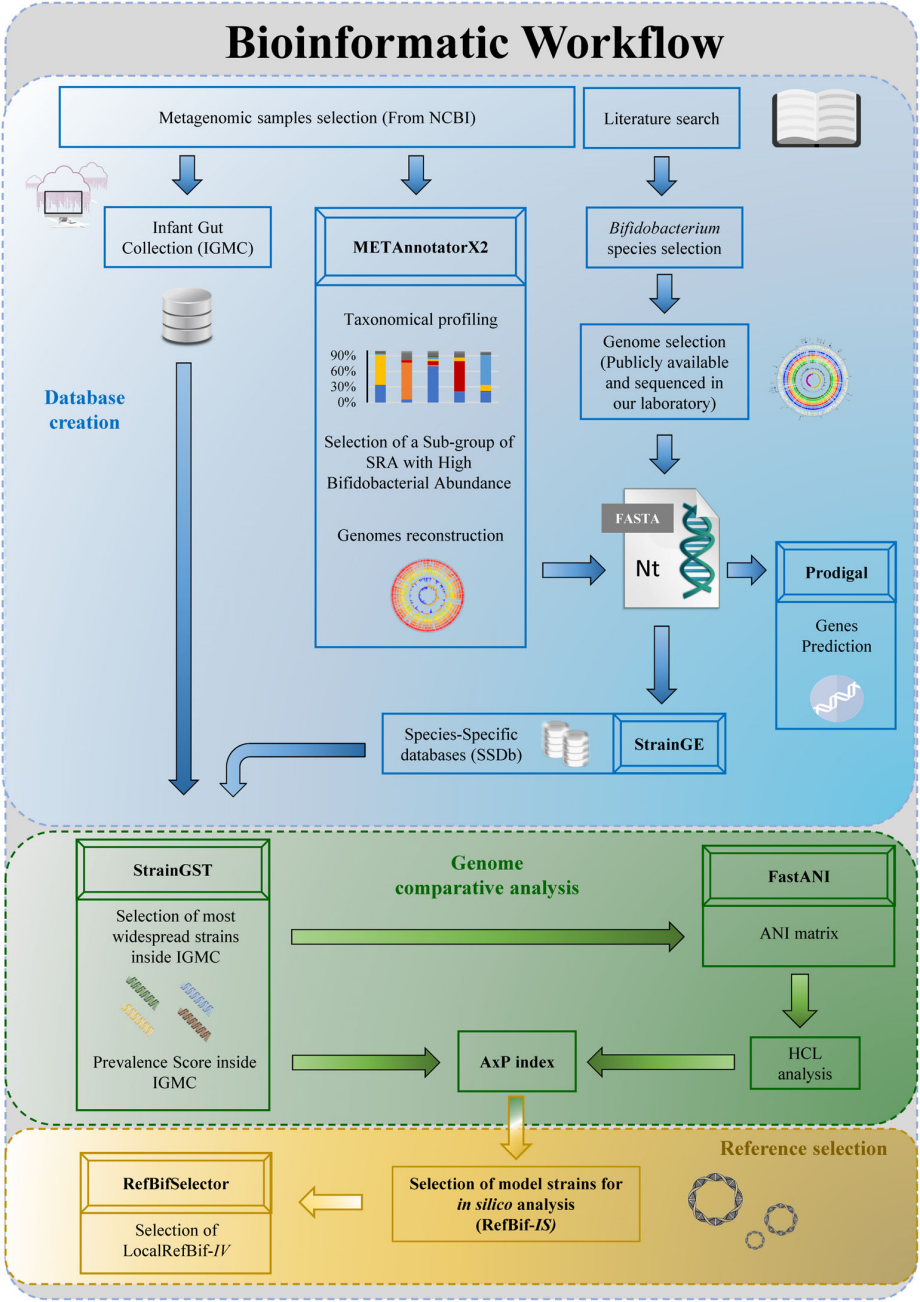
Bioinformatics workflow. This figure depicts a schematic workflow concerning the main bioinformatics steps performed. The workflow is divided into three main blocks, outlining steps for data recovery, their analysis and the creation of databases (blue block), the analyses on selected genomes (green block), and the final selection of RefBif‐IS and LocalRefBif‐IV (yellow block).

In order to analyse the ecological distribution of the most representative bifidobacterial strains of the infant gut microbiota, we established an infant faecal sample database called Infant Gut Microbiota Collection (IGMC), consisting of metagenomic data sets of faecal samples from individually selected healthy infants, ranging in age from a few days to 3 years, sequenced through Illumina‐based technology in order to avoid any bias related to different sequencing platforms (Figure 1) (Table S1) Table 1. Based on these criteria, a total of 1664 datasets covering 17 different cohorts from various geographical areas were selected and analysed through METAnnotatorX2 (Milani et al., 2021) (Table S1). Additional information regarding the IGMC can be found in the Supplementary Text S1.

**TABLE 1:** SRA metadata summary report

Bioproject	SRA count	% of IGMC (%)	Age (average days)	Country	Count	Prevalence in IGMC (%)
PRJEB12669	14	0.84	544	Europe	593	35.64
PRJEB24771	233	14.00	183	USA	554	33.29
PRJEB32135	65	3.91	252	Malawian	233	14.00
PRJEB6456	199	11.96	239	New Zealand	125	7.51
PRJNA287207	2	0.12	90	Canada	65	3.91
PRJNA290380	394	23.68	545	South Africa	56	3.37
PRJNA322188	32	1.92	44	Italy	24	1.44
PRJNA339914	5	0.30	90	China	14	0.84
PRJNA345144	125	7.51	362			
PRJNA352475	14	0.84	120			
PRJNA422569	3	0.18	730			
PRJNA473126	56	3.37	235			
PRJNA475246	60	3.61%	90			
PRJNA524703	306	18.39	156			
PRJNA542703	14	0.8	45			
PRJNA549787	56	3.37	113			
PRJNA557731	86	5.17	30			

Abbreviation: IGMC, Infant Gut Microbiota Collection.

The IGMC dataset was scrutinized to define the most abundant and prevalent bifidobacterial species, representing the bifidobacterial ‘core’ infant gut microbiota. Specifically, this bifidobacterial core community was defined by selecting those bifidobacterial taxa showing a prevalence of >10% and an average abundance of >0.5%, so as to consider only those species playing a relevant role in the infant gut microbiota (Turroni et al., 2021). In this context, *B. adolescentis*, *B. bifidum*, *B. breve*, *B. catenulatum*, *B. longum, B. dentium* and *B. pseudocatenulatum* were shown to be the most representative *Bifidobacterium* species across the IGMC, confirming previously published data (Arboleya et al., 2016; Duranti et al., 2017; Laursen et al., 2021; Milani et al., 2015; Turroni, Milani, Duranti, Ferrario, et al., 2018) (Figure 1) (Table S2) (Details are provided in the Supplementary Text S1).

Subsequently, datasets showing a *Bifidobacterium* genus average relative abundance >10% were selected to reconstruct the genomes of bifidobacterial strains corresponding to the above‐identified most prevalent species. The threshold of 10% average relative abundance in bifidobacterial composition was selected to obtain enough genetic material (i.e. reads) to reconstruct (near complete) genomes. This procedure allowed the reconstruction of 239 bifidobacterial metagenomes assembled genomes (MAGs), including 105, 47, 30, 19, 16, 13, and nine chromosomal sequences belonging to the *B. longum*, *B. bifidum*, *B. breve*, *B. pseudocatenulatum*, *B. dentium*, *B. adolescentis*, and *B. catenulatum* species, respectively (Table S3). These MAGs comprise the dominant strains of a bacterial species along with fragments of sequences derived from other strains belonging to the same bacterial species if present at relatively high abundance.

So, MAGs are microbial reconstructed genomes representative of the bacterial species encompassing the microbiomes of the samples assayed.

These reconstructed MAGs, combined with 965 publicly available bifidobacterial genomes and 93 bifidobacterial strains that had previously been isolated and sequenced within the context of the current study, were then used to generate Species‐Specific Databases (SSDbs), in total encompassing 1297 bifidobacterial chromosomes of the abovementioned key microbial players of the infant gut microbiota (Figure 1) (Table S3). This database, which covers the highest genomic diversity available for each bifidobacterial species, was built to evaluate the prevalence of bifidobacterial strains within the previously created IGMC (Tables S3 and S4).

To evaluate the distribution of bifidobacterial strains among the IGMC datasets, a StrainGST‐based profiling analysis was conducted, resulting in the identification of 209, 76, 70, 48, 47, 21 and 19 strains belonging to *B. longum*, *B. bifidum*, *B. breve*, *B. adolescentis*, *B. pseudocatenulatum*, *B. dentium* and *B. catenulatum* species, respectively, for a total of 490 bifidobacterial genomes showing a prevalence >0.1% across the IGMC datasets (Figure 1) (Table S5). These 490 bifidobacterial genomes were subjected to ANI analysis to evaluate genome similarity, and the obtained ANI data were subsequently used to perform a hierarchical clustering analysis (HCA) (Liang et al., 2018; OriginLab, 2020), resulting in a series of species‐specific HCL trees. (Supplementary excel File S1) (Details are provided in the Supplementary Text S1).

**FIGURE 2:**
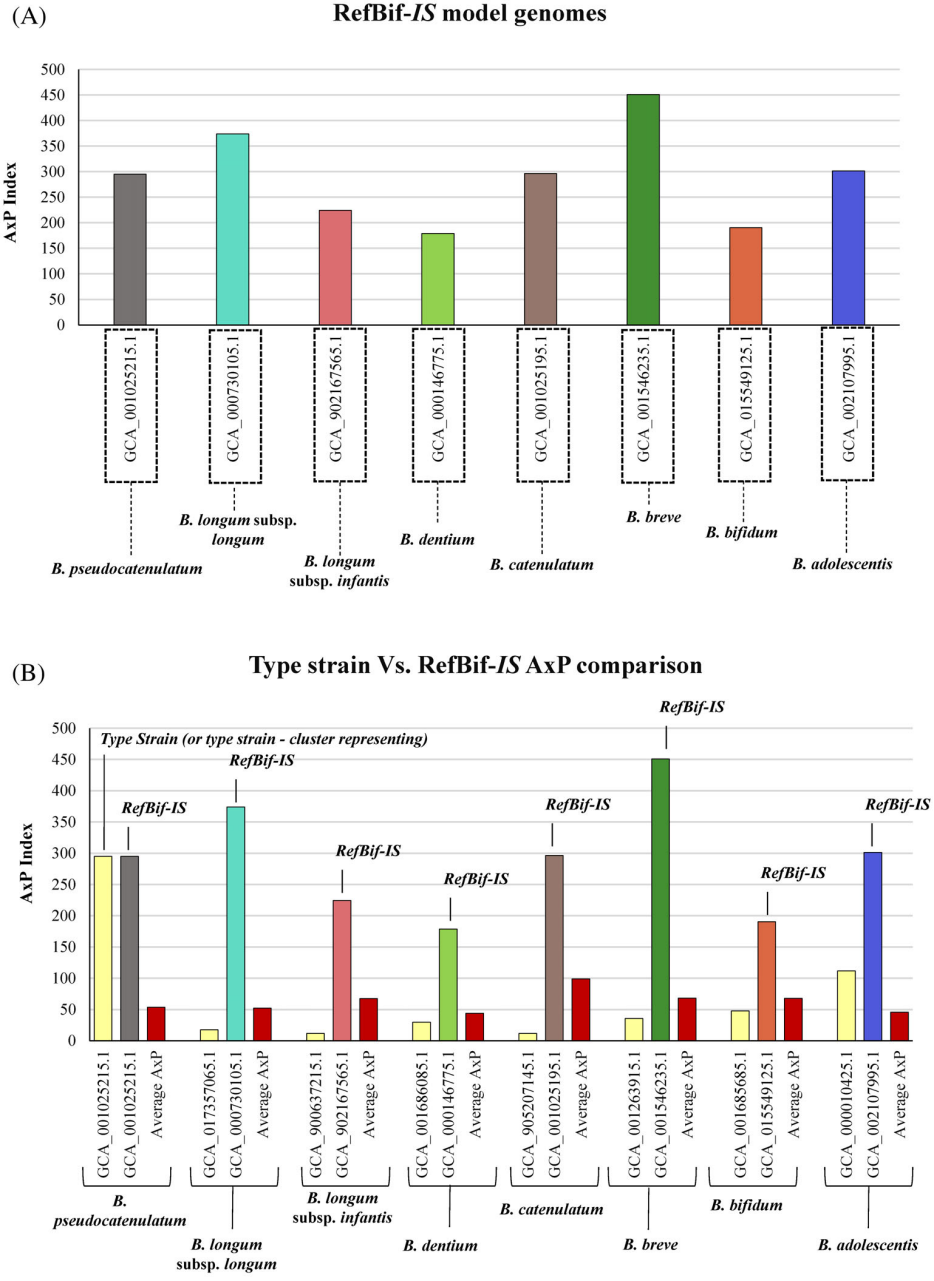
Model strain for in silico and in vitro analyses. Panel (A) reports, in the *x* axis, the optimal reference strains for in silico analyses identified for each bifidobacterial species typical of the infant gut microbiota, while the *y* axis shows the AxP index associated with each RefBif‐*IS*. Panel (B) reports a comparison between the type strain versus RefBif‐*IS* and their AxP values, with the average AxP of the total HCL as reference.

Finally, strain prevalence and ANI data were integrated into a specific index score, that is, the Average × Prevalence index (A × P index), as described in the Experimental Procedures. This score led to the selection of eight reference strains with the highest A × P scores for each species‐specific HCL tree, therefore considered to represent the optimal Reference Bifidobacterial strains for in silico analyses (RefBif‐*IS*) (Supplementary excel File S1) (Figures 1 ad 2) (supplementary text S1). Therefore, these eight reference bacterial strains can be considered the most ecologically and genetically representative bifidobacterial strains of the infant gut microbiota (Table 2). Intriguingly, with the exception of *B. pseudocatenulatum* JCM 1200^T^, all other former Type Strains (or type strain cluster representing from StrainGE analysis) *B. longum* subsp. *longum* ATCC 15707^T^, *B. longum* subsp. *infantis* ATCC 15697^T^, *B. dentium* DSM 20436^T^, *B. catenulatum* DSM 16992^T^, *B. breve* ATCC 15700^T^, *B. bifidum* JCM 1255^T^ and *B. adolescentis* ATCC 15703^T^ show a much lower A x P value than the RefBif‐*IS* (Figure 2). This result suggests that these bifidobacterial type strains do not accurately represent the genetic content or ecological distribution of each bifidobacterial species, as also recently suggested (Figure 2) (Lapage et al., 1992). Although this approach allocates equal weight to all genes, this was done to normalize the results, considering the prevalence as genetic adaptation favourable to the environment, thus assuming the weight of all genes simultaneously. In conclusion, these A × P values emphasize that the suitability of the former physiologically defined type strains to act as model organisms for each species should mainly be based on the genetic make‐up of microbial species, and thus on their genomic variability (Supplementary excel File S1) (Figure 2).

**TABLE 2:** RefBif‐*IS* selected as optimal reference strains

Species	Strain	GCA
*Bifidobacterium adolescentis*	LMG 10734	GCA_002107995.1
*Bifidobacterium bifidum*	1001283B150225_161107_H11	GCA_015549125.1
*Bifidobacterium breve*	GED8481	GCA_001546235.1
*Bifidobacterium catenulatum*	JCM 1194	GCA_001025195.1
*Bifidobacterium dentium*	ATCC 27679	GCA_000146775.1
*Bifidobacterium longum* subsp*. longum*	1‐5B	GCA_000730105.1
*Bifidobacterium longum* subsp*. infantis*	*B.longum*_ssp_infantis_5	GCA_902167565.1
*Bifidobacterium pseudocatenulatum*	JCM 1200	GCA_001025215.1

### RefBifSelector: A tool for biobank screening

While RefBif‐*IS* represents a valuable genetic‐driven approach to identify suitable reference strains for each infant bifidobacterial species, it may be that the indicated strains are not publicly available and/or easy to retrieve/obtain. We therefore considered the possibility to expand the selection also to those strains that are available in non‐public repositories (e.g. local microbial collections) in order to identify phylogenomically related alternatives that labs may use as alternative reference models. For this reason, we developed a tool named RefBifSelector, which allows a rapid screening of local biobanks for bifidobacterial strains that are closely related to the optimized reference strains defined by RefBif‐*IS* (https://probiogenomics.unipr.it/cmu/) (Figure S1). This tool requires the bacterial genome sequences in fasta format to be screened as input (Query) and provides the best alternative to the reference RefBif‐*IS* genomes as output. This evaluation is performed through ANI score in conjunction with the use of the average percentage of positive scoring matches (PPOS). While ANI analysis investigates the nucleotide identity between genome pairs, PPOS is a score retrieved from Blastp analysis that uses the following formula to compare the translated amino acid sequences: [(number of identical matches) + (number of similar matches)]/(alignment length). Therefore, a final score equal to the value of ANI * Average_PPOS was obtained, considering a minimum score threshold equal to 9600 (corresponding to an ANI and Average_PPOS of 98). Strains below this minimum threshold cannot be assessed adequately similar to RefBif‐*IS* and cannot be considered suitable choices.

In order to validate this bioinformatic pipeline, genomes of bifidobacterial strains belonging to our local biobank were submitted as input to the RefBifSelector tool. Specifically, among the 34 screened strains, *B. bifidum* PRL2010, *B. longum* subsp. *longum* 39B, *B. breve* 1895B and *B. pseudocatenulatum* 1896B displayed the best choice in terms of ANI and Average PPOS value with respect to the RefBif‐*IS* references, thus representing the LocalRefBif‐*IV* (in vitro), while *B. catenulatum*, *B. adolescentis*, *B. dentium* and *B. longum* subsp. *infantis* were excluded from the analysis since no strains of human origin were available in the bifidobacterial strains repository that we employed (Table S6).

### In vitro validation of the identified LocalRefBif‐*IV*

Optimal bacterial models should allow the efficient investigation of microbe–microbe and host–microbe interactions, including those occurring in complex microbial populations.

In this context, to further corroborate the biological suitability of the strains identified through the RefBifSelector tool, we assayed the ability of the identified LocalRefBif‐*IV* and the other members of the infant gut microbiota using an in vitro model. Therefore, the four bifidobacterial strains identified as LocalRefBif‐*IV* among our local bifidobacterial strain collection were cultivated in a simulated infant intestinal environment. Additionally, the respective former bifidobacterial type strains for *B. pseudocatenulatum*, *B. longum, B. breve* and *B. bifidum*, that is, LMG10505, LMG13197, LMG13208 and LMG11041 and four strains of our local biobank identified as the most genetically dissimilar to the RefBif‐*IS* for *B. pseudocatenulatum*, *B. longum*, *B. breve* and *B. bifidum*, that is, 1052B, 209B, 31L and 324B, were cultivated to evaluate and compare their growth performances with that of the four bifidobacterial strains predicted as LocalRefBif‐*IV*, that is, 1896B, 39B, 1895B and PRL2010. Specifically, each selected strain was inoculated in batch culture systems using a complex medium mimicking the infant intestinal environment (Macfarlane et al., 1998; Zihler Berner et al., 2013) together with a healthy 3‐year‐old infant‐derived gut microbial community previously stabilized through a continuous fermentation model. Cultivation samples taken at 6, 12, and 24 h were collected for each strain, together with a single control sample, that is, the inoculated stabilized infant gut microbial community. Subsequently, the microbial DNA extracted from each condition was subjected to shallow shotgun sequencing (Hillmann et al., 2018; Milani et al., 2021), generating a total of 919,824 reads with an average of 48,411 reads per sample, reduced to a total of 695,150 reads and an average of 36,586 reads per sample after quality‐filtering (Table S7).

**FIGURE 3:**
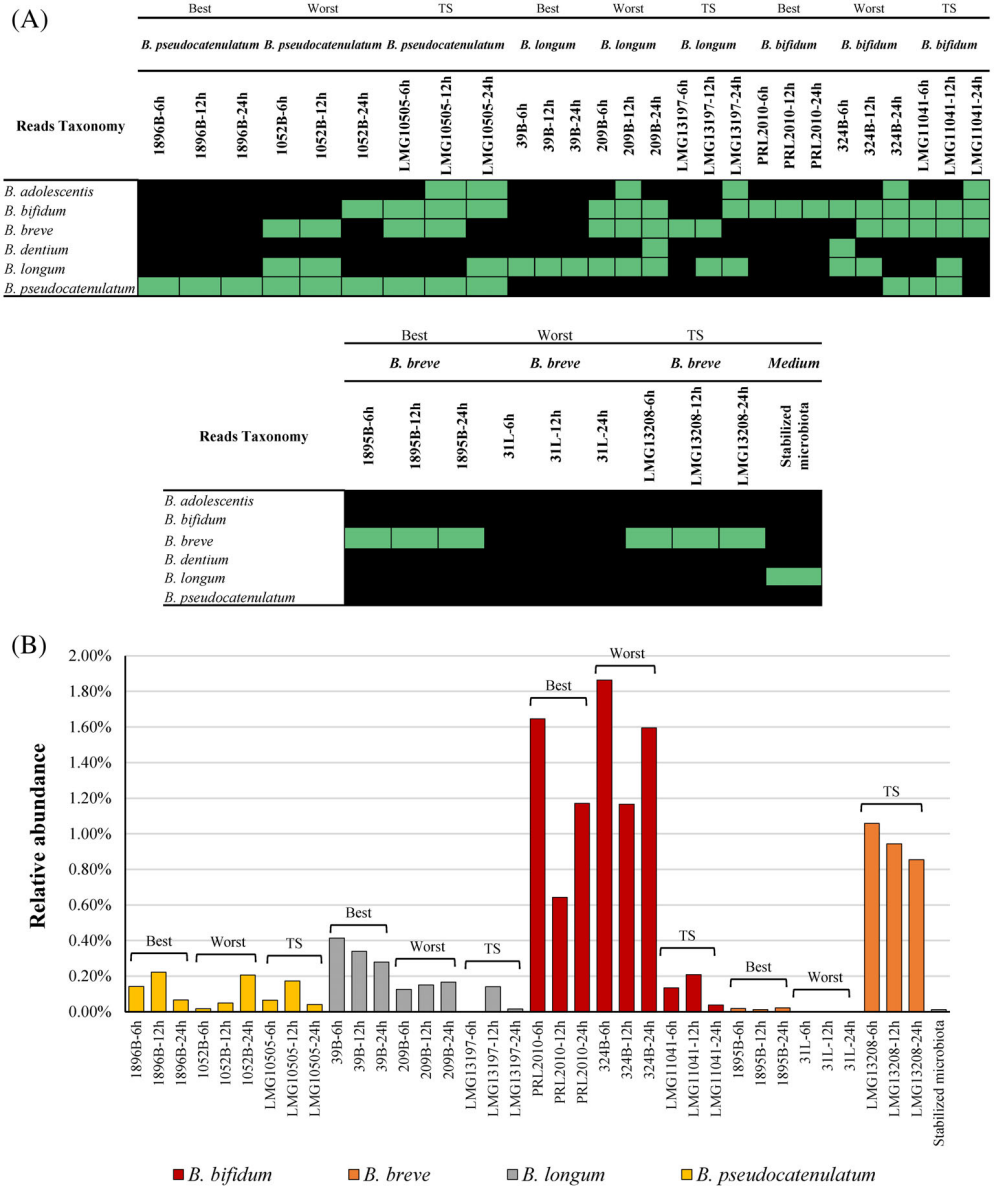
Detection of LocalBifRef‐*IV* in a simulated intestinal environment. Panel (A) depicts the presence analysis of tested bifidobacterial strains with >0.01% relative abundance when grown in a simulated intestinal environment. Panel (B) reports the relative abundance reached by the same bifidobacterial strains after 6, 12 and 24 h of fermentation. Each strain was given the adjective of worst (sub‐optimal candidate), best (LocalRefBif‐*IV*) and TS (former type strain).

Taxonomic profiling at the species‐level highlighted the presence of traces of *B. longum* in the control sample (Table S7) (Figure 3). The relative abundance of this species in the stabilized infant gut microbial community corresponds to <0.01%. Thus, the presence of *B. longum* species in the control sample represents background noise that is not expected to affect downstream bioinformatic analyses.

Remarkably, taxonomic profiles of the obtained microbial cultures inoculated with LocalRefBif‐*IV* strains revealed the ability of these selected bifidobacterial strains to actively grow, as evidenced by a relative average abundance of 0.14% for *B*. *pseudocatenulatum*, 0.34% for *B*. *longum* and 1.15% for *B*. *bifidum* between the three tested time points, with the exception of *B. breve* 1895B with only a 0.02% of average relative abundance, values that correspond to the average relative abundance of the *Bifidobacterium* genus in the human gut microbiota, typically <2% (at genus level) (Do Nam et al., 2011; Odamaki et al., 2016) (Table S7) (Figure 3). In contrast, the former bifidobacterial type strains of these species included in the analysis showed lower colonization performances, as evidenced by a relative average abundance of 0.093% for *B*. *pseudocatenulatum*, 0.052% for *B*. *longum* and 0.13% for *B*. *bifidum* between the three tested time points (Tables S6 and S7) (Figure 3). An exception to this pattern was observed for the B. *breve* LMG13208 type strain, which showed an average relative abundance of 0.952% (Tables S6 and S7) (Figure 3). In addition, the sub‐optimal model bifidobacterial strains that we used in our trials showed an average relative abundance that was somewhere between the former type strains and the optimal model strains as suggested by LocalRefBif‐*IV*, except for sub‐optimal *B*. *breve* 31L, which showed no growth both at 6, 12 and 24 h (Tables S6 and S7) (Figure 3).

Notably, these results confirm that the ecologic and genomic‐based approach proposed for the choice of optimal microbial models allows the identification of strains able to colonize and persist in complex microbial communities, allowing the study of the microbe–microbe interplay.

### LocalRefBif‐*IV* as suitable model strains for in vitro bifidobacteria–host interaction experiments

**FIGURE 4:**
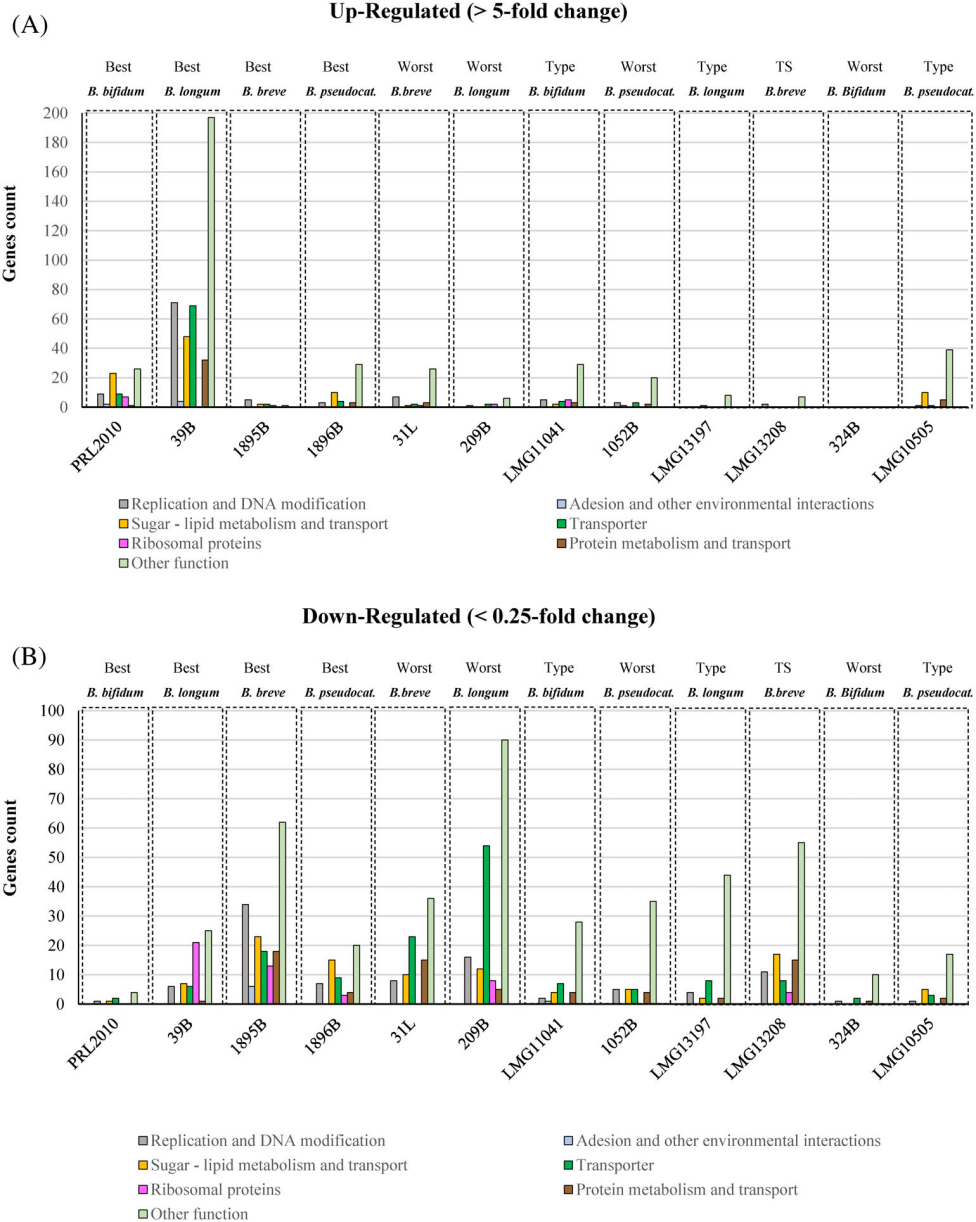
Transcriptomics of LocalRefBif‐*IV* strains incubated on Caco2/HT29‐MTX cell monolayers. Panel (A) reports a selection of up‐regulated (>5‐fold change) genes, subdivided in family function, of the four selected LocalRefBif‐*IV* strain placed in contact with Caco2/HT29‐MTX cell monolayers. Panel (B) reports a selection of down‐regulated (<0.25‐fold change) genes, subdivided in family function, of the four selected LocalRefBif‐*IV* strains incubated on Caco2/HT29‐MTX cell monolayers.

The ability to interact with the host is another important feature required for an optimal reference model strain representing a commensal bacterium residing in the human gut. Thus, in order to validate the latter characteristic for the bifidobacterial strains suggested by LocalRefBif‐*IV*, we performed in vitro trials involving human cell lines where *B. bifidum* PRL2010, *B. longum* subsp. *longum* 39B, *B. breve* 1895B and *B. pseudocatenulatum* 1896B strains were placed in contact. In addition, for comparison purposes, we included the four strains identified in our local bifidobacterial biobank as the most dissimilar to the RefBif‐*IS* of *B. bifidum* and *B longum* subsp. *longum*, *B. breve* and *B. pseudocatenulatum* along with the respective deposited type strains (Table S8). The cross‐talk features of these strains with the human host were then inspected by transcriptomics experiments (Table S8). In this context, each of the 12 strains was incubated on co‐cultured Caco2/HT29‐MTX cell monolayers. Transcriptomic profiles, acquired by RNAseq experiments, were compared with those obtained from the same strains cultivated in batch on DMEM liquid media in the same incubation conditions without any contact with human cell monolayers, representing the reference conditions (Table 3) (Figure 4).

**TABLE 3:** RNA expression profiling summary

Strains growth on human cell monolayer versus control						
Tested strain	Fold change					
	>3	>5	<0.25	<=0.5		Type
	Gene count				Up (>5)/down (<0.25)	
PRL2010	207	77	8	79	9.6250	Best
39B	732	421	66	183	6.3788	Best
1895B	26	11	174	420	0.0632	Best
1896B	91	49	58	303	0.8448	Best
31 L	179	40	92	265	0.4348	Worst
209B	52	11	185	591	0.0595	Worst
LMG11041	159	48	46	178	1.0435	TS
1052B	80	29	54	398	0.5370	Worst
LMG13197	61	9	60	178	0.1500	TS
LMG13208	46	9	110	438	0.0818	TS
324B	79	0	14	223	0.0000	Worst
LMG10505	187	56	28	146	2.0000	TS

*Note*: Best: optimal local reference strain; TS: type strain of relative species; Worst: sub‐optimal local strain.

*B. bifidum* PRL2010 showed 77 overexpressed (fold change > 5) and only eight downregulated (fold change < 0.25) genes, resulting in extensive activation of genes involved in the metabolism of carbon sources (24) and trans‐membrane transport (11), as well as replication and signalling (10) along with two genes putatively involved in extracellular adhesion (Table S8) (Table 3) (Figure 4). Furthermore, *B. longum* subsp. *longum* 39B data showed 421 up‐regulated and 66 down‐regulated genes, thus being extensively affected by the interaction with human cells. In detail, *B longum* subsp. *longum* 39B showed activation of genes encoding the biosynthetic machinery for Tad pili, 55 genes involved in the metabolism of carbohydrates and lipids along with a notable number, that is, 76, of transporter‐encoding genes and 66 genes with a putative role in transcriptional regulation (Table S8). In contrast, *B. breve* 1895B and *B. pseudocatenulatum* 1896B showed a strong transcriptional downregulation of genes, with a ratio between up‐regulated and down‐regulated (in the test situation and compared with control) equal to 0.0632 and 0.8448, respectively (Table 3).

These results were compared with those obtained from the corresponding type strains and for the four strains locally available and identified as the most genetically diverging from RefBif‐*IS* (Table S6). Intriguingly, the sub‐optimal strain *B. bifidum* 324B showed no up‐regulated genes and only 14 down‐regulated genes, highlighting the limited response of this strain to contact with human cells, while *B. bifidum* type strain LMG11041 showed a total of 94 genes with a ratio of 1.01 between up‐regulated and down‐regulated. In contrast, LocalRefBif‐*IV B. bifidum* PRL2010, with a total of 85 genes and a ratio of 9.63 between up‐regulated and down‐regulated, resulted to be extensively stimulated by the contact with the human cell monolayers (Table 3) (Figure 4). Similar results can be observed analysing LocalRefBif‐*IV B. longum* subsp. *longum* 39B that, with a total of 489 genes and a ratio of 6.38 between up‐regulated and down‐regulated genes, showed a strong transcriptional activation, which is higher than the original type strain *B. longum* subsp. *longum* LMG13197 and the sub‐optimal strain *B. longum* subsp. *longum* 209B (Table 3) (Figure 4). Instead, all analysed strains of *B. breve* showed a ratio between up/down regulated genes ranging between 0.06 and 0.4, highlighting a strong down‐regulation of genes in *B. breve* species when in contact with human cell monolayers (Table S8) (Table 3) (Figure 4).

Altogether, these data confirm that *B. bifidum* PRL2010, *B. longum* subsp. *longum* 39B, *B. breve* 1895B and *B. pseudocatenulatum* 1896B represent suitable model strains, as expected, due to their genetic affinity with the RefBif‐*IS*.

Remarkably, co‐cultivation with human cell lines confirmed that the identification of model microbes based on ecological and genomic data allows the definition of optimal reference model strains for the investigation of the intricate network of interactions existing between microbes and their host.

## CONCLUSIONS

In this study, bifidobacterial species representing the ‘core’ infant gut microbiota were exploited as model taxa for the development of a novel approach to determine optimal reference strains based on strain tracking and comparative genomics investigations. Based on the integration of ecological, genomic and functional data, this approach was successfully employed to identify reference strains as research models that are representative of their corresponding species. In this regard, we employed a multi‐omics approach that demonstrated how the proposed RefBif‐*IS* reference strains can be used to carry out various in vitro experiments, that are not limited to the taxonomical classification alone. Moreover, to facilitate the use of these enhanced model reference strains, a user‐friendly tool named RefBifSelector was developed to enable the screening of local strain biobanks to identify the strains phylogenetically closer to the RefBif‐*IS* references, referred to as LocalRefBif‐*IV*.

In this context, a model microorganism should be represented by an optimal reference strain for each bacterial species in terms of containing the genetic capabilities of the members of that bacterial species to establish within its natural ecological niche. Therefore, when we applied this genomic‐approach to identify reference strains for certain key bifidobacterial species that are naturally residing in the human gut, we decided to validate the novel identified bifidobacterial type strains identified through LocalRefBif‐*IV* strains by performing in vitro analyses of their genetic capabilities to interact with other members of the gut microbiota as well as with the human host. Moreover, comparisons of the functional results with those retrieved for the actual recognized type strains for the bifidobacterial species here assayed and additional sub‐optimal candidates highlighted that the LocalRefBif‐*IV* possess all the characteristics to be considered excellent reference strains candidates for in vivo experiments, as well as being the most genetically similar to RefBif‐*IS*, as expected from the collected ecological and functional data.

## CONFLICT OF INTEREST

The author declares that there is no conflict of interest that could be perceived as prejudicing the impartiality of the research reported.

## Supporting information


**Appendix S1:** Supporting Information

**Figure S1:** Supporting Information

**Supplementary File 1:** ANI comparison for the selection of model strains for in silico analysis, named also RefBif‐IS, through resulting AxP index.

**Table S1:** SRA metadata and taxonomical profiling results obtained through the use of METAnnotatorX2 software.

**Table S2:** Average relative abundance and prevalence of Bifidobacterium species in the analysed publicly available shotgun metagenomics datasets (SRA).

**Table S3:** List of genomes used as input for the creation of the SSDbs (through the use of strainGST software).

**Table S4:** Additional information about Bifidobacterium genomes publicly available on online NCBI genomes repository.

**Table S5:** StrainGST results and analysis

**Table S6:** Comparison between local available strains and RefBif‐IS throught RefBifSelector

**Table S7:** Taxonomical profiling of LocalRefBif‐IV fermentation in simulated intestinal environment obtained through the use of METAnnotatorX2 software.

**Table S8:** RanSeq results of tested strains, grown singularly with and without contact with human cells line.

## Data Availability

Raw sequences of shallow shotgun sequencing coupled with RNA sequencing data are accessible through the Bioproject PRJNA844015 on NCBI. RefBifSelector software is downloadable from the https://probiogenomics.unipr.it/cmu/web page.
