# Supplementary material for: Designation of optimal reference strains representing the infant gut bifidobacterial species through a comprehensive multi‐omics approach
Source: Environ Microbiol. 2022 Oct 7;24(12):5825–39. doi: 10.1111/1462-2920.16205 (PMC10092070; doi:10.1111/1462-2920.16205)
Supplement: Supplementary file 1 — Appendix S1: Supporting Information [file EMI-24-5825-s009.docx]

**Establishment of the Infant Gut Microbiota Collection (IGMC).**

The ecological distribution of the most representative bifidobacterial strains in the infant gut microbiota was identified exploiting all the publicly available (on NCBI repository) infant fecal shotgun metagenomic datasets obtained through Illumina sequencing technology (Figure_S1). Quality-filtering of reads associated with these retained samples generated 106,201,091 filtered reads with an average of 63,822 reads per sample (Table_S1).

**Development of bifidobacterial species-specific databases (SSDbs).**

To profile the ecologic distribution of the most representative bifidobacterial strains occurring in the infant gut microbiota, we developed Species-Specific Databases (SSDbs) covering the highest genomic variability available for each bifidobacterial species.

Although with a prevalence of 4.39% and an average relative abundance of 0.4%, also *B. dentium* was included in downstream analyses since it has been reported as an important bifidobacterial species of the infant gut microbiota (Table_S2).

Assembled genome sizes were comparable to the average genome length expected for each species based on previous literature and exhibited an Average Nucleotide Identity (ANI) value >94 % with respect to other conspecific members. The SSDbs also included strains of non-human origin as negative controls, which, as intended, were not identified in the retrieved strain-level profiles. Further information on publicly available genomes used in this study can be found in supplementary materials (Table_S4).

The SSDbs were subjected to the removal of redundant strains using a specific function of the strainGE suit that automatically retains only one of the genomes with more than 99% k-mers profile identity and subsequently employed for strain profiling (Figure_S1).

**Evaluation of bifidobacterial strains prevalence in the IGMC and identification of the optimal reference model strains for *in silico* analyses.**

The 490 bifidobacterial genomes profiled through StrainGST, which represent the most ecologically relevant bifidobacterial strains of the infant gut microbiota in terms of prevalence and relative abundance, were submitted to ANI analysis performed through the FastANI software to evaluate genome similarity (Supplementary_excel_File_1). As reported in literature, ANI values >94 % represents the breakpoint value suggesting that those strains belong to the same species, while an ANI value of 100% indicates identical strains.
